# 55484 Dual activation of CAR and Nrf2 improves the efficacy: toxicity ratio of cyclophosphamide and doxorubicin-based treatment of TNBC

**DOI:** 10.1017/cts.2021.653

**Published:** 2021-03-30

**Authors:** Sydney Stern, Dongdong Liang, Linhao Li, Ritika Kurian, Caitlin Lynch, Scott Heyward, Ajoke Kareem, Young Chun, Charles Hong, Fengtian Xue, Hongbing Wang

**Affiliations:** 1 University of Maryland Baltimore School of Pharmacy; 2 NCATS; 3 BIOIVT; 4 University of Maryland Baltimore School of Medicine

## Abstract

ABSTRACT IMPACT: Triple negative breast cancer (TNBC) affects 10-20% of women with breast cancer and is biologically more aggressive than other subtypes. The novel compound we have developed, DL7076, would give clinicians a vital strategy to improve the commonly used cyclophosphamide (CPA) and doxorubicin (DOX) regimen in the treatment of TNBC. OBJECTIVES/GOALS: The objective of this research project is to develop a novel compound which can activate both 1) the constitutive androstane receptor (CAR) and subsequently enhance the CYP2B6-mediated activation of CPA, and 2) the nuclear factor erythroid- related factor-2 (Nrf2) leading to the cardiomyocyte protection from DOX-associated cardiotoxicity. METHODS/STUDY POPULATION: Following the identification of the compound candidate, DL7076 was evaluated for tissue specific induction of CAR and Nrf2 using qPCR, western blot analysis, and luciferase reporter assays.

Further, we have developed a multicellular coculture model incorporating human primary hepatocytes for metabolism, TNBC spheroids as the target, and cardiomyocytes as a side target of DOX. We have investigated the anticancer effects of CPA/DOX on TNBC cells and the toxic effects on cardiomyocytes with/without a CAR-Nrf2 activator, in a multicellular environment where hepatic metabolism is well-retained. RESULTS/ANTICIPATED RESULTS: We found that our dual activator of CAR and Nrf2, DL7076, exhibits tissue specific induction of CAR and Nrf2. Inclusion of DL7076 in combination with the CPA/DOX regimen improves anticancer efficacy, through the subsequent increase in the formation of the active CPA metabolite. With the addition of DL7076, DOX-mediated off-target cardiotoxicity was markedly reduced.

Lastly, utilizing the novel coculture system with human primary hepatocytes, TNBC spheroids, and cardiomyocytes, the inclusion of DL7076 to the CPA/DOX regimen shows decreased spheroid viability and improved cardiomyocyte viability and function. DISCUSSION/SIGNIFICANCE OF FINDINGS: Our findings suggest that DL7076 can facilitate DOX/CPA containing regimens by increasing CAR-mediated metabolism and subsequent CPA bioactivation while selectively protecting cardiomyocytes from DOX-induced toxicity. This research is expected to translate our basic scientific findings into therapeutic interventions for women with TNBC.

